# Dynamic Behavior of Thermally Affected Injection-Molded High-Density Polyethylene Parts Modified by Accelerated Electrons

**DOI:** 10.3390/polym14224970

**Published:** 2022-11-16

**Authors:** Ales Mizera, Lovre Krstulovic-Opara, Nina Krempl, Michaela Karhankova, Miroslav Manas, Lubomir Sanek, Pavel Stoklasek, Alen Grebo

**Affiliations:** 1 Faculty of Applied Informatics, Tomas Bata University in Zlin, 760 05 Zlin, Czech Republic; 2 Faculty of Electrical Engineering, Mechanical Engineering and Naval Architecture, University of Split, HR-21000 Split, Croatia; 3 Department Polymer Engineering and Science, Montanuniversitaet Leoben, 8700 Leoben, Austria

**Keywords:** mechanical properties, infrared thermography, high-density polyethylene, injection-molded technology, temperature stability, radiation cross-linking

## Abstract

Polyethylenes are the most widely used polymers and are gaining more and more interest due to their easy processability, relatively good mechanical properties and excellent chemical resistance. The disadvantage is their low temperature stability, which excludes particular high-density polyethylenes (HDPEs) for use in engineering applications where the temperature exceeds 100 °C for a long time. One of the possibilities of improving the temperature stability of HDPE is a modification by accelerated electrons when HDPE is cross-linked by this process and it is no longer possible to process it like a classic thermoplastic, e.g., by injection technology. The HDPE modified in this way was thermally stressed five times at temperatures of 110 and 160 °C, and then the dynamic tensile behavior was determined. The deformation and surface temperature of the specimens were recorded by a high-speed infrared camera. Furthermore, two thermal methods of specimen evaluation were used: differential scanning calorimetry (DSC) and thermogravimetric analysis (TGA). The result of the measurement is that the modification of HDPE by accelerated electrons had a positive effect on the dynamic tensile behavior of these materials.

## 1. Introduction

Polymer materials are increasingly finding their place in applications where, until recently, classic materials, such as steel, glass, ceramics, etc., were used. In terms of volume, the most used polymers are from the polyolefin group. This group mainly includes polyethylenes (low-density polyethylene and high-density polyethylene). Polyethylenes are characterized by excellent workability, low weight and excellent chemical resistance. The main advantage of polyethylene products is low weight while maintaining relatively good mechanical properties. On the other hand, the disadvantage is low temperature stability, which prevents the use of polyethylenes in engineering applications where the operating temperature is usually well above 100 °C [1,2,3,4,5,6,7].

The most common methods of polyethylene processing are extrusion, injection, blowing and compression molding. By choosing the processing technology, we influence the morphology of the material, which affects the resulting mechanical properties of the final product [1]. One of the most widespread methods of processing HDPE is injection molding, which is one of the most precise manufacturing technologies, capable of repeated production of quality pieces in large series. This technology has spread, e.g., in the field of automotive, aerospace, electronics, etc. Thanks to its low price and its high productivity, there is no need for further modifications to its surfaces, dimensions, or shapes [8,9]. Currently, there are many ways to process HDPE by injection technology so that the productivity is as high as possible, but at the same time, the stability of the operation is maintained, where attention is paid to high dimensional accuracy and surface quality [10,11,12,13,14,15,16].

HDPE is commonly used as a packaging material for food, laboratory equipment, and other components due to its properties, such as easy processing, low weight and durability. The methods of recycling this material are also successfully used, and the recyclate is used again in the production of primary products. However, only a few scientific works deal with this issue. Most professional studies are devoted to the recycling of polypropylene (PP) or polyethylene terephthalate (PET) [3,17,18,19].

Expanding the application possibilities of HDPE to other industrial areas is possible; however, it is necessary to modify this material, especially in order to achieve higher temperature stability. The mechanical properties are also significantly worse, compared to engineering (construction) polymers (e.g., polyamides and polycarbonates). Various additives or fillers (e.g., carbon fibers or nanofillers) are used, which often significantly improve the properties of HDPE [20,21,22,23,24,25,26]. Based on the results from thermal analyses, such as differential scanning calorimetry DSC and thermogravimetrical analysis TGA, it is possible to ascertain whether modified HDPE is suitable for engineering applications or not. Another possibility of testing is the use of spectral analyses, e.g., Fourier Transform Infrared Spectroscopy FTIR, from which the degree of degradation of this material can be detected, both after modification and after temperature loading. The material should withstand long-term exposure to the temperature, for which it was designed, for the duration of its service life and without noticeable degradation or wear [27,28,29,30,31,32].

One of the possibilities of HDPE modification is the use of radiation cross-linking by accelerated electrons. This modification of HDPE proved to be very effective, especially from the point of view of temperature stability. The advantage is that this type of modification does not need any cross-linking agent, and the modification is carried out only on the final product, which makes it possible to modify only part of the product, and the rest can have the properties of the original material. Despite increasing demands on product quality, there is no need to reach for a new, more powerful material immediately and therefore a new tool/injection mold, but you can use the existing one and only modify the product. Another important factor is the price of the modification by accelerated electrons, which varies in industrial applications in the range of EUR 1–2/1 kg depending on the complexity of the product and the quantity of the order. Logistics and storage costs must be added to this price. However, it is necessary to carefully consider all the pros and cons and perform all the necessary tests to ensure that the resulting modified product is safe for its application while still being competitive [33,34,35,36,37,38].

A huge disadvantage of products made from HDPE modified by radiation cross-linking by accelerated electrons is that the material loses its unique property, namely thermoplasticity. This leads to the fact that cross-linked HDPE can no longer be further processed as a thermoplastic, which means that a suitable method of recycling such modified materials needs to be found [39,40,41,42,43]. Ahmad and Rodrigue wrote in their review about the possibilities of recycling radiation cross-linked polyethylenes. The result of the study is that there is currently no cheap and energy-efficient method of processing polyethylenes modified in this way [44]. Manas et al. [45,46] in their studies added crushed rHDPE to pure HDPE and investigated the workability of these mixtures and the subsequent mechanical properties. This reprocessing method is cheap and simple, and the decrease in mechanical properties is minimal [45,46].

The disadvantage is the impossibility of remelting the modified polyethylene by accelerated electrons; on the other hand, there is an advantage, namely a cross-linked structure that resists higher temperatures. Materials modified in this way can be used even in applications with increased temperatures and withstand without them any damage. The use of PE modified by this method in engineering applications is possible, but these materials need to be thoroughly tested. This study is focused on the dynamic tensile behavior of HDPE modified by accelerated electrons. The deformation of the test body was recorded with a high-speed infrared camera, and the surface temperature before rupture was evaluated.

Many studies deal with both the static and dynamic tensile or compressive properties of various materials. Deformations are also evaluated in various studies with a recording of the surface temperature by an infrared camera. Each of the studies evaluates different materials, especially under a static tensile load [47,48,49,50,51,52,53,54,55,56,57,58]. However, no study was found regarding dynamic uniaxial straining using infrared thermography.

During dynamic tensile stress, the material is deformed, and the place is heated (especially the place of plastic deformation), which subsequently causes damage to the product. Using this technique, it is possible to predict damage before it occurs. This study deals with surface temperature scanning during dynamic tensile stress in modified HDPE products after thermal stress.

## 2. Materials and Methods

In a previous study, it was found that the modification of HDPE by accelerated electrons had a significant effect on the temperature aging of injected components [38]. It was further shown that the changes in mechanical properties proved to be significant under impact stress [38]. Therefore, in the present study, we focused on the dynamic behavior of temperature-loaded components that were produced by injection molding technology.

### 2.1. Material and Specimen Preparation

The same material was used in this study as in the previous study [38]. It is a commercially produced high-density polyethylene (HDPE) with the trade name HDPE 25055 E, which was supplied by DOW (Midland, MI, USA). Due to its very narrow molecular weight distribution and melt flow index of 25 g/10 min (190 °C/2.16 kg), this polymer is easily processed by injection molding technology, and it also stands out for its mechanical properties and high-gloss and high-surface finishing. The material supplier recommends using this material in applications such as housewares, food containers and toys.

**Figure 1:**
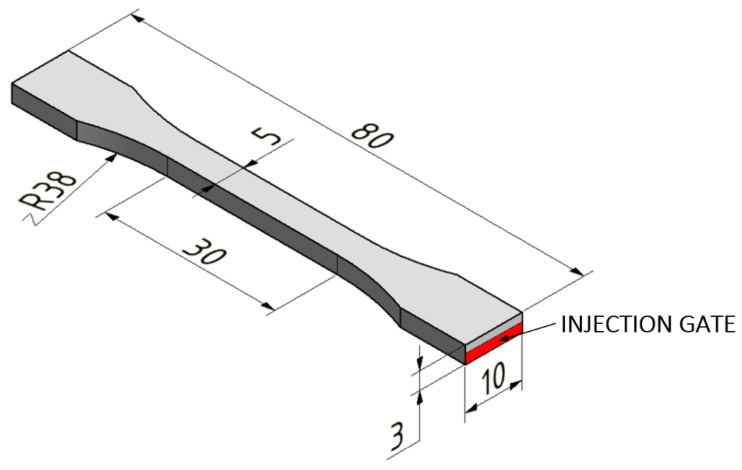
Shapes and dimensions of tested specimens. Adapted from [38], MDPI, 2022.

The test samples were produced by an injection molding machine, Arburg Allrounder 170 U, with screw diameter 15 mm (Loßburg, Germany). Process parameters, which can be seen in Table 1, were set as optimal conditions according to the recommendation of the material’s manufacturer. The shapes and dimensions of the specimens that were tested (Figure 1) were governed by the CSN EN ISO 527-1 [38].

### 2.2. Radiation Cross-Linking by Accelerated Electrons

The produced test specimens were modified by accelerated electrons. Based on previous studies, four radiation doses (99, 132, 165 and 198 kGy) were selected, which showed the best properties (mechanical properties and temperature stability). The process of radiation cross-linking of HDPE test specimens took place under normal atmospheric conditions at room temperature. The modification of the test specimens was carried out in cooperation with the company BGS Beta-Gamma-Service, located in Germany. In detail, the source of electrons was a hot cathode made from wolfram, and then these electrons were accelerated in a strong electric field and a high vacuum of a Rhodotron high-voltage accelerator, which presented the maximum energy of 10 MeV (Tongeren, Belgium). The adequate radiation dose was determined by a Nylon FTN 60-00 dosimeter (Far West Technology, Inc., Goleta, CA, USA). The analysis of absorbed radiation dose by the dosimeter was performed with a Genesys 5 spectrophotometer (Thermo Fisher Scientific, Waltham, MA, USA) in accordance with the ASTM 51261 standard [38].

### 2.3. Tempereature Aging

**Figure 2:**
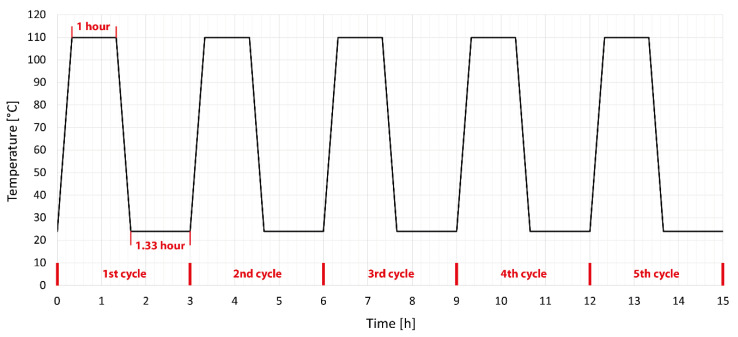
Setting temperature aging cycle of HDPE under the melting point [38].

**Figure 3:**
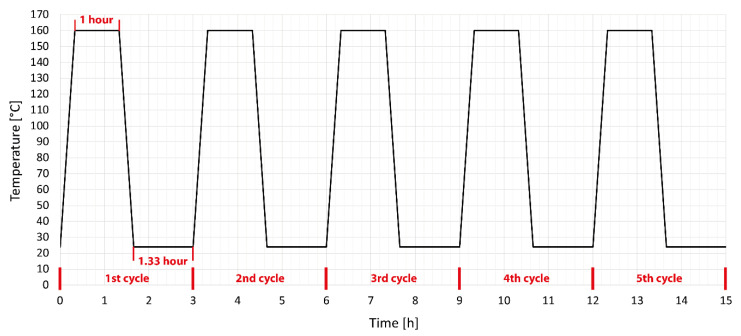
Setting temperature aging cycle of HDPE above the melting point [38].

The produced test specimens were divided into four groups. The first group consisted of test specimens that were not modified in any way or subjected to temperature stress. All measurement results were compared to this group of specimens. The second group consisted of test specimens modified by accelerated electrons without temperature load. The third group consisted of test specimens modified by accelerated electrons, which were subjected to five cycles in a temperature chamber at a temperature of 110 °C; the detailed temperature profile is shown in Figure 2. This temperature profile was chosen so that the set temperature did not reach the melting temperature of unmodified HDPE and moved within the safe limits of short-term use of unmodified HDPE. The fourth group consisted of test specimens modified by accelerated electrons, which were temperature-stressed by five cycles in a temperature chamber at a temperature of 160 °C; the detailed temperature profile is shown in Figure 3. This profile was chosen with regard to the short-term use of modified HDPE specimens by accelerated electrons, which they can withstand several times under this temperature load. The test specimens were tempered according to predefined temperature cycles between two 6 mm-thick steel plates with a nonstick surface. This method of heating was chosen to avoid possible deformations due to different temperatures on the bottom and top of the specimens. At the end of the cycle, the test specimens were sorted and stored for further testing.

### 2.4. Dynamic Tensile Test with Infrared Thermography Record

A Uniaxial tensile servohydraulic INSTRON 8801 (Instron, Norwood, MA, USA) test machine was used for dynamic testing of modified HDPE specimens. Measurements were carried out according to the ISO 527 standard at standard ambient conditions (23 °C and 60% relative humidity). Displacement controlled tension test with crosshead speed of 284 mm/s was set. Three measurements from each specimen were tested, and their ultimate tensile strength values were evaluated. Conditioning was taken for five days at a temperature of 23 °C and relative humidity of 60%. Arithmetic mean and standard deviation were used in all figures [59].

The deformation process of the test specimens was evaluated using infrared (IR) thermography. Acquisition was carried out by a high-speed InSb middle-wave IR thermal camera Flir SC5000 (FLIR Systems, Wilsonville, OR, USA) cooled at cryogenic temperatures. The frame rate was 320 Hz with a thermal sensitivity of 0.02 K sensitive cooled middle-wave InSb detector. Plastic deformation was observed, and surface temperature was evaluated during the dynamic tensile test [59].

### 2.5. Differential Scanning Calorimetry (DSC)

The differential scanning calorimetry (DSC) was carried out with a measurement de-vice, Type DSC 1 (Mettler Toledo GmbH, Wien, Austria). The specimens were prepared with a scalpel and were always taken from the same position on each injection molding specimen so that they were approximately the same in shape, size and weight. The DSC analysis was used to investigate the melting and crystallization behavior of the specimens. The average specimen weight was approx. 10 mg.

The measurements were carried out according to ISO 11357-4 in heating and cooling mode with a heating rate of 10 K/min or a cooling rate of 20 K/min in the temperature range from room temperature (RT) to 190 °C. The thermal DSC program included the first heating from 20 °C to 190 °C with a heating rate of 10 °C/min, the first cooling from 190 °C to 20 °C with a cooling rate of 20 °C/min and the second heating from 20 °C to 190 °C with a heating rate of 10 °C/min. All experiments were performed under an N_2_ atmosphere with a gas flow of 50 mL/min.

### 2.6. Thermogravimetric Analysis (TGA)

The thermal properties of the tested specimens (approx. 0.8500 g of each) were investigated using a thermogravimetric analyzer prepASH Series 340 (Precisa Gravimetrics AG, Switzerland) operating at temperatures of up to 1000 °C equipped with a built-in high-performance analytical balance of 0.0001 g. Weighing curves were detected and recorded over time for each individual test specimen. The following temperature profile was employed for the analysis: 20 °C, 6 °C/min to 200 °C, 3 °C/min to 320 °C, 1 °C/min to 500 °C and 3 °C/min to 650 °C (hold 60 min). All measurements took place in the presence of air.

## 3. Results

Based on a previous study [38], four modified HDPEs by accelerated electrons at irradiation doses of 99, 132, 165 and 198 kGy were selected. These four doses of radiation proved to be the most suitable from the point of view of mechanical properties, as well as from the point of view of temperature stability. The field of impact or dynamic mechanical stress proved to be a very interesting area of study, where it was shown that HDPE modified by accelerated electrons could have better properties than unmodified HDPE.

In this study, we focused on the dynamic behavior of HDPE modified by accelerated electrons in tension. The course of dynamic loading was recorded by a high-speed infrared camera, which recorded the change in surface temperature, especially in the area of the plastic deformation of the specimens.

### 3.1. Dynamic Tensile Test with Infrared Thermography Record

For better clarity, the tested specimens were divided into four groups; their abbreviations and descriptions are shown in Table 2. The first group contained only unmodified HDPE (pure HDPE), which served in this study only as a standard against which the results of the other three groups of specimens (modified and temperature-stressed) were compared.

**Figure 4:**
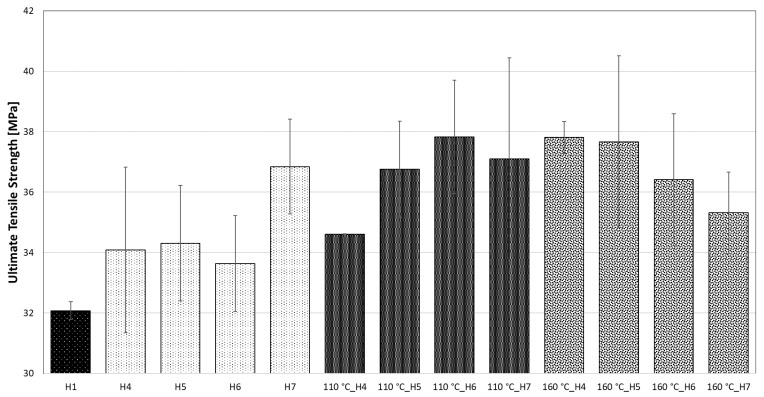
Graphic display of dynamic tensile test results.

**Figure 5:**
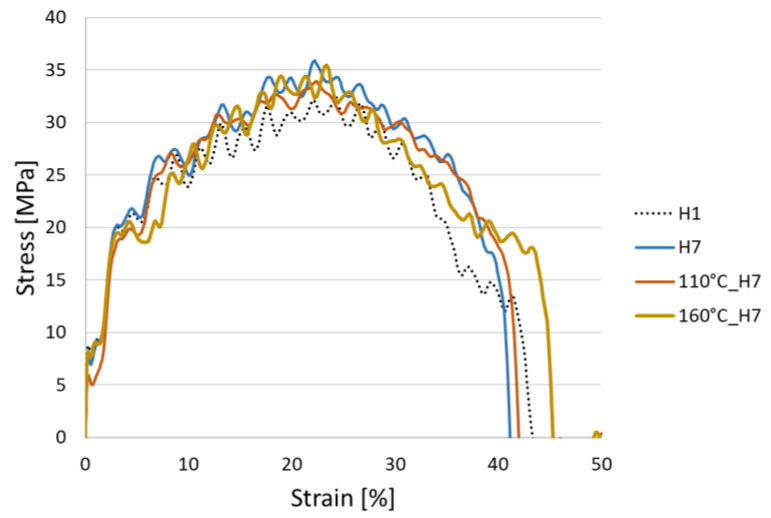
Stress–strain curves.

Figure 4 graphically grows the statistically evaluated values of the ultimate tensile strength of all measured specimens. The unmodified HDPE achieved the lowest ultimate tensile strength of 32.1 ± 0.3 MPa. For all other modified specimens, whether the temperature was affected or not, a higher ultimate tensile strength was achieved. However, the variability of the measured values for modified HDPE was significantly larger than for unmodified HDPE. The reason may be the inconsistency of the internal network during the modification of HDPE by accelerated electrons. During dynamic stress (fast loading), the material does not have the possibility to relax (adapt) to the external stress, thus every defect in the 3D internal network contributes to the reduction and variability of the ultimate tensile strength. The ultimate tensile strength of modified HDPE with radiation doses of 99, 132 and 165 kGy (group 2) increased by 6%, with a radiation dose of 198 kGy up to 15% compared to unmodified HDPE (group 1). The highest increase in ultimate tensile strength was achieved for specimen 110 °C_H6, when after five times with a temperature load at 110 °C there was an increase of 12.5% compared to specimen H6. This increase could have occurred as a result of postcrystallization or post-cross-linking of H6 specimens under the influence of the temperature of 110 °C. In the last group of specimens, which were temperature-loaded five times at 160 °C, the ultimate tensile strength gradually decreased from 37.8 ± 0.5 MPa to 35.3 ± 1.4 MPa with increasing radiation doses. This reduction probably occurred due to the release of internal stress and incipient thermooxidation processes. However, the results of the measurement of dynamic properties show that even after five times the temperature load at 110 and 160 °C, the dynamic tensile properties did not deteriorate; on the contrary, there was a substantial improvement. Figure 5 shows representative stress–strain curves from each group of specimens. The profile of the curves was similar; however, the ultimate tensile strength value was different. The lowest ultimate tensile strength value was recorded for sample H1 (group 1); in contrast, the ultimate tensile strength values for the other displayed specimens were higher, which was statistically evaluated in Table 3.

**Figure 6:**
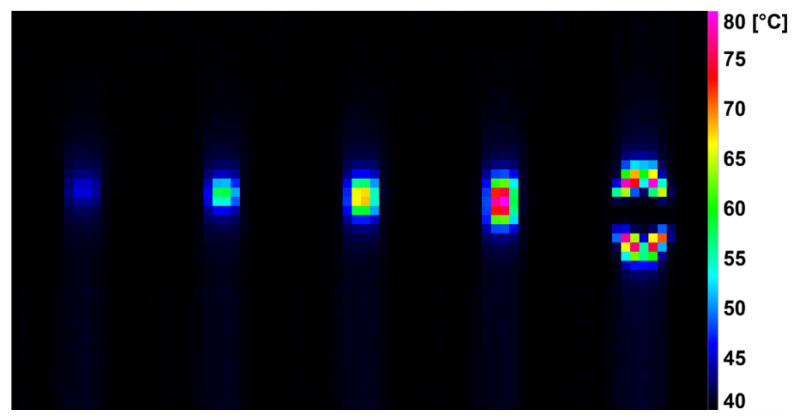
Deformation process of H1 evaluated using IR thermography.

**Figure 7:**
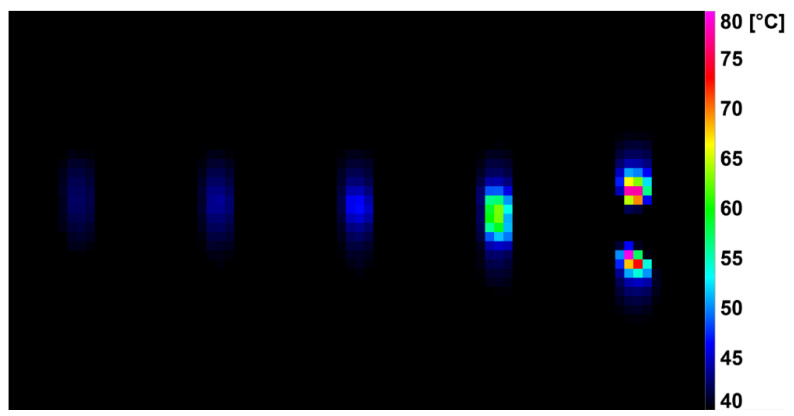
Deformation process of H7 evaluated using IR thermography.

**Figure 8:**
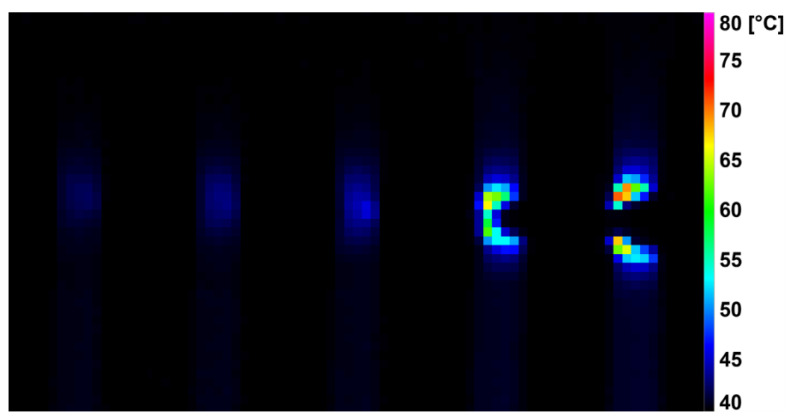
Deformation process of 110 °C_H7 evaluated using IR thermography.

**Figure 9:**
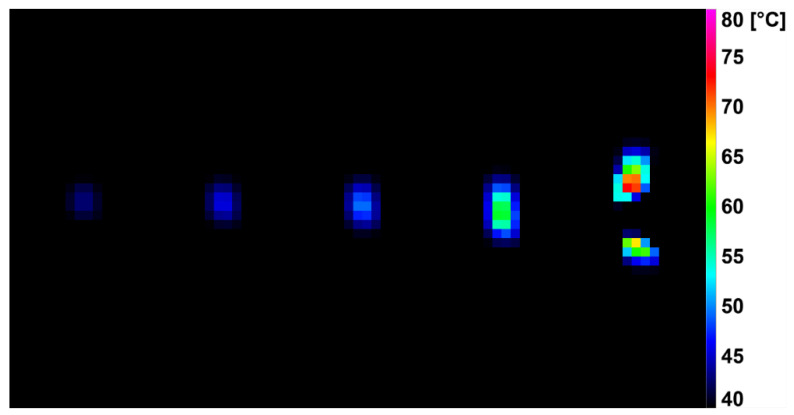
Deformation process of 160 °C_H7 evaluated using IR thermography.

During the dynamic tensile test, the deformation was detected by a high-speed infrared camera, which recorded the current surface temperature of the tested specimens. The difference in surface temperature values at the beginning of the measurement and just before breaking the specimens are shown in Table 3. It was noted that the surface temperature of the unmodified samples increased by 40.0 ± 4.0 °C during the dynamic tensile test. For the modified HDPE (group 2), there was a reduction in the surface temperature rise during the test by 30–43%. An even greater decrease (by 41–57%) in the increase in surface temperature was achieved by the modified HDPE subjected to a temperature load of 110 °C (group 3). The last group was modified HDPE after temperature loading at 160 °C (group 4), where a decrease in surface temperature increase of 15–32% was measured. Figure 6, Figure 7, Figure 8 and Figure 9 show deformation processes using thermography; specimens H1, H7, 110 °C_H7 and 160 °C_H7 were selected (representatives from each group of specimens). The actual surface temperatures before the test specimen rupture are shown here. The sample H1 reached the highest surface temperature during dynamic loading.

### 3.2. Differential Scanning Calorimetry (DSC)

**Figure 10:**
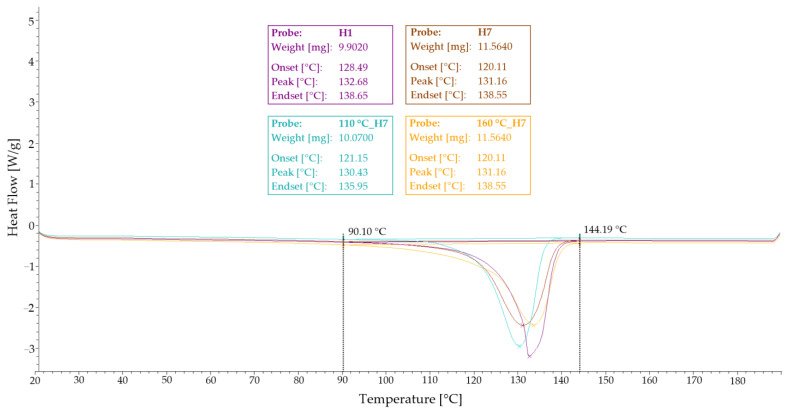
DSC measurement—1st heating of selected specimens from each group.

Differential scanning calorimetry (DSC) was chosen as one of the methods of accurately evaluating the history of the influence of the material due to processing or other processes that can affect the thermal history of the specimens. Values at the 1st heating, 1st cooling and 2nd heating were recorded. The peak temperature was evaluated based on these values. Table 4 shows all these values. The measured values in the individual groups differed slightly, but the thermographic results were not confirmed by this test. Both the injection process itself and the modification and temperature loading affected the HDPE specimens. Figure 10 shows a record of DSC measurements for specimens H1, H7, 110 °C_H7 and 160 °C_H7 (a representative of each group of specimens). The differences were minimal.

### 3.3. Thermogravimetric Analysis (TGA)

**Figure 11:**
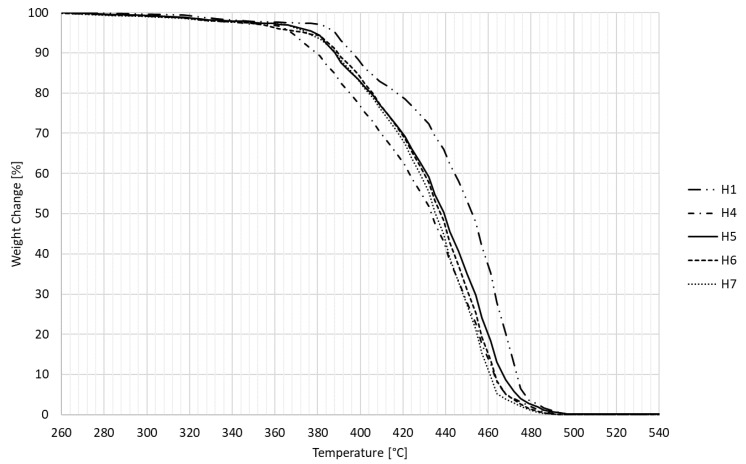
Degradation curves of nonmodified and modified HDPE specimens.

**Figure 12:**
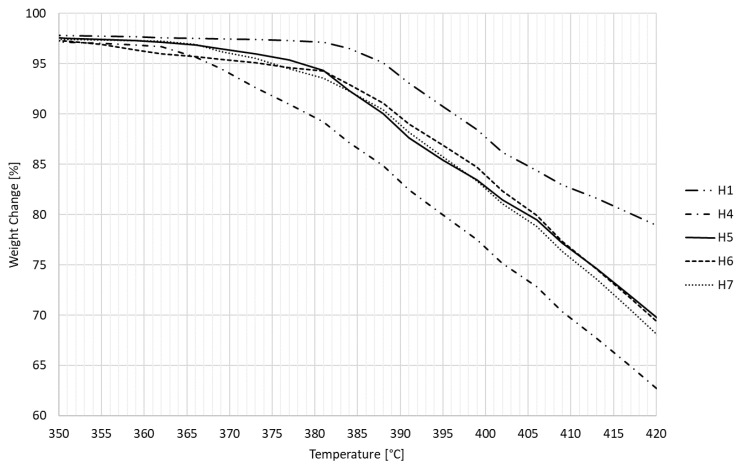
Degradation curves of nonmodified and modified HDPE specimens in detail.

Thermogravimetric analysis (TGA) was chosen as the last test, which shows the progress of material degradation based on weight loss at increasing temperatures. Figure 11 shows a recording from TGA. The unmodified HDPE (H1) began to degrade rapidly at a temperature of 380 °C, while the other modified HDPEs already began to decrease at a temperature of 365 °C. In detail, this area is captured in Figure 12. Specimens from groups 3 and 4 were not measured by this method; no change in degradation temperature was expected, which could occur due to a five-time temperature load at 110 and 160 °C. The degradation temperature was slightly affected by the degree of modification (network density), which increased with an increasing radiation dose up to the maximum applied radiation dose of 198 kGy. However, all tested samples experienced complete vaporization at the same temperature of 490 °C.

## 4. Discussion

In a previous study [38], it was already proven that the modification of HDPE by accelerated electrons has a significant effect on static mechanical properties and especially on temperature stability. Modified HDPE can withstand several times the temperature up to 160 °C, while unmodified HDPE immediately melts. Even after this multiple-temperature load, the static tensile properties did not deteriorate [38]. Based on these findings, a study was conducted that dealt with the dynamic tensile loading of HDPE modified by accelerated electrons, which was then subjected to a five-time temperature load at temperatures of 110 and 160 °C. The results of the dynamic tests show that the modified HDPE had better properties than unmodified HDPE by up to 15%. It was also confirmed that temperature loading at 110 and 160 °C did not lead to the deterioration of behavior under dynamic tensile loading. The detection of deformation with a high-speed infrared camera turned out to be very profitable. Here, substantial differences emerged for unmodified HDPE (group 1), modified HDPE (group 2), modified HDPE after a temperature load of 110 °C (group 3) and modified HDPE after a temperature load of 160 °C (group 4). The highest surface temperature value was recorded for unmodified HDPE, and the lowest surface temperature was recorded for sample group 3. It will be appropriate to investigate this area more deeply in the future to see if this trend is also confirmed for other materials modified by accelerated electrons. Furthermore, DSC was used, which could confirm this finding, but from the measured results, it was only possible to state that both the production technology (injection) and the modification or temperature loading had a minimal effect on the thermal history of the specimens. However, the trend from the measurement of the surface temperature of the specimens was not confirmed. TGA was chosen as the last test, which showed a slight change in the degradation temperature of the unmodified HDPE compared to other modified HDPE specimens. Rapid weight loss occurred for all tested samples between 360 and 490 °C under a normal atmosphere.

## 5. Conclusions

It was found that the modification of HDPE by accelerated electrons had a significant effect on the dynamic tensile behavior of both HDPE specimens, which were temperature-unstressed, and HDPE specimens, which were temperature-stressed by five-time temperature loading at 110 or 160 °C. Furthermore, after a five-time temperature load at 110 °C (group 3), the surface temperature was reduced by up to 57% compared to the unmodified HDPE (group 1), while there was no deterioration of the properties under dynamic tensile loading. DSC and TGA showed specific characteristics of the modified properties but did not directly lead to the clarification of why the modified HDPE specimens after a temperature load of 110°C experienced such a significant decrease in the surface temperature during dynamic loading compared to the unmodified HDPE. In the future, we will focus on clarifying this phenomenon, and we will focus in more detail on the entire process of dynamic loading, and we will repeat the experiment for more materials from the polyolefin group.

## Figures and Tables

**Table 1:** Injection molding parameters.

**Processing Conditions**	**170 U**
Injection Rate (mm/s)	40
Injection Pressure (MPa)	60
Holding Pressure (MPa)	40
Holding Time (s)	30
Cooling Time (s)	30
Mold Temperature (°C)	40
**Plastic Unit Temperature Bands**	**170 U**
Zone 1 (°C)	180
Zone 2 (°C)	190
Zone 3 (°C)	195
Zone 4 (°C)	200

**Table 2:** Abbreviations and description of used specimens for dynamic tensile test.

Group	Abbreviation	Description
1	H1	Nonmodified HDPE
2	H4	Modified HDPE by irradiation dose 99 kGy
2	H5	Modified HDPE by irradiation dose 132 kGy
2	H6	Modified HDPE by irradiation dose 165 kGy
2	H7	Modified HDPE by irradiation dose 198 kGy
3	110 °C_H4	H4 after temperature load at 5 × 110 °C
3	110 °C_H5	H5 after temperature load at 5 × 110 °C
3	110 °C_H6	H6 after temperature load at 5 × 110 °C
3	110 °C_H7	H7 after temperature load at 5 × 110 °C
4	160 °C_H4	H4 after temperature load at 5 × 160 °C
4	160 °C_H5	H5 after temperature load at 5 × 160 °C
4	160 °C_H6	H6 after temperature load at 5 × 160 °C
4	160 °C_H7	H7 after temperature load at 5 × 160 °C

**Table 3:** Evaluated data from the dynamic tensile test with recorded surface temperature differences.

Specimen	Ultimate Tensile Strength [MPa]	Surface Temperature Difference [°C]
H1	32.1 ± 0.3	40.0 ± 4.0
H4	34.1 ± 2.7	26.4 ± 6.6
H5	34.3 ± 1.9	28.2 ± 0.4
H6	33.6 ± 1.6	22.8 ± 5.4
H7	36.8 ± 1.6	25.5 ± 4.7
110 °C_H4	34.6 ± 0.1	17.0 ± 2.5
110 °C_H5	36.8 ± 1.6	23.4 ± 3.9
110 °C_H6	37.8 ±1.9	17.8 ± 2.5
110 °C_H7	37.1 ± 3.3	17.5 ± 10.3
160 °C_H4	37.8 ± 0.5	29.4 ± 7.2
160 °C_H5	37.7 ± 2.8	33.8 ± 8.1
160 °C_H6	36.4 ± 2.2	32.7 ± 3.7
160 °C_H7	35.3 ± 1.4	27.0 ± 4.7

**Table 4:** Evaluated data from DSC.

Specimen	Peak Temperature at 1st Heating [°C]	Peak Temperature at 1st Cooling [°C]	Peak Temperature at 2nd Heating [°C]
H1	132.68	117.39	130.81
H4	133.80	114.71	131.49
H5	134.17	111.76	134.27
H6	131.35	113.17	131.17
H7	131.16	111.98	130.96
110 °C_H4	131.97	115.41	130.84
110 °C_H5	131.72	114.10	131.56
110 °C_H6	133.42	111.68	133.04
110 °C_H7	130.43	113.35	130.30
160 °C_H4	134.58	114.22	131.81
160 °C_H5	134.87	113.04	132.71
160 °C_H6	133.74	112.80	132.12
160 °C_H7	133.55	111.77	132.12

## Data Availability

The data presented in this study are available on request from the corresponding author.
